# Atlanta Society of Medicine

**Published:** 1891-11

**Authors:** 


### ATLANTA SOCIETY OF MEDICINE.—OCT. 6, 1891.

This was the regular evening for the report of cases.

DR. HARDON reported an interesting case of twin pregnancy with placenta prævia, which he saw in consultation. When he arrived there was profuse hæmorrhage, large pool of blood under the patient, face blanched and pulse weak. Upon examination, found the os dilated to about size of a half-dollar, and could feel placenta with finger. Under chloroform passed whole hand into womb. After passing edge of placenta, hand encountered feet, and at the same time a head, and he at once diagnosed twin pregnancy. Tried to pull down feet but they would not pass the head of other child; so he again passed his hand and turned the second child, when delivery was accomplished without further trouble.

DR. COOPER reported a similar case, in which there was one very large placenta (twice the usual size), and a very long cord.

DR. CRAWFORD said that some weeks ago a child, one week old, was brought to him with ophthalmia neonatorum. He ordered silver nitrate, ten grains to the ounce of water, and a solution of bichloride of mercury, 1–10,000, with instructions to wash the eyes frequently with latter, and to return to his office daily for application of the former. Instead, the mother washed the eyes with the silver solution, and dropped the bichloride in the eyes. We would expect injury to eyes from such treatment, but child returned in one week and eyes were well.

DR. CRAWFORD also reported a case of natural absorption of cortical cataract in child fourteen years old. Lens almost entirely absorbed. Only about fifty such cases on record.

DR. HUTCHINS reported case of chancre of finger in a man, aged forty-eight. Six weeks before seeing him patient had little sore on finger, said to have begun as a boil. When seen there was a raw sore, size of a quarter, with some suppuration. For long time could get no satisfactory history. At last patient said he had acted part of midwife in labor case, and it was afterwards learned that husband of woman had sore on penis. Eight weeks later patient had macular eruption on abdomen, and papular on forehead. Sore is not healing and seems to be phagedenic.

DR. ELKIN said he had known of two cases of chancre of fingers, both in physicians. Both were contracted in making examination of women suffering from secondary manifestations and mucous patches in the vagina. In each case the infection took place in a "hang-nail." Reported further, some cases of syphilitic paralysis. In one case he saw the primary sore, and in three weeks eruption appeared; but the patient became careless of his treatment, and in four or five months left it off entirely. Then developed iritis, for which he took treatment for a few months, and then stopped. In two years had left hemiplegia. Dr. Elkin thought that if treatment had been properly carried out complications would not have occurred. He would impress the importance of a positive diagnosis before beginning general treatment, so as to be able more fully to impress the patient with the necessity of following the treatment a long time and continuously.

DR. COOPER told of two cases of tertiary syphilis, in which there had been neither diagnosis nor treatment. Patient, aged thirty-six, gave no history of syphilis but said that six years before he had sore on penis, which physician said was due to irritation from clothing. Upon examination, found cicatrix on foreskin, supposed to be that of old chancre, and another just above knee. On posterior aspect of leg and ankle were eight sloughing ulcers, varying in size and depth, and surrounded with livid skin. Put patient on iodide potash, and applied local treatment to ulcers. Kept him in bed two weeks. Healthy granulations sprang up rapidly, and in thirty days all ulcers had cicatrized. Also reported enlarged testicle from syphilis, complicated with hydrocele. Tapped the hydrocele. Was a distinct cavity in upper part of testicle. Tumor was eight inches in circumference and of stony hardness. Diagnosis: Carcinoma or tertiary syphilis. Referred case to Dr. Elkin, who thought it syphilitic. Used anti-syphilitic treatment, and in two weeks tumor was reduced in size one-half. Lost sight of patient for long time, when he came to office for severe headache. This supervened about two weeks ago upon discontinuance of iodide potash. There were also vertigo and disposition to fainting. Resumed the iodide. Testicle now normal, with exception of slight hydrocele.

DR. MCRAE showed specimens of bone removed from substance of brain in injury to head. There were entire absence of shock and other symptoms usually seen in such cases. Skull was crushed in just posterior to coronal suture. As the depressed bone was denuded, thought best to trephine. No bad symptoms at any time, and patient made rapid recovery. Further reported case of lateral operation for stone. Patient, aged three years. Four or five soft phosphatic stones situated in anterior urethra; one impacted in neck of bladder. Removed former with forceps through meatus. For the latter did lateral operation and washed out bladder. Child well in twelve days.

DR. GRANDY reported case of calculus removed from fossa navicularis, through meatus. Stone phosphatic and about diameter of No. 30 sound. Patient, aged 42, and very hysterical.

DR. L. P. KENNEDY reported a premature birth. The seven-months child, though feeble, was doing well, and had fair promise of living.

